# The T3SS of *Shigella*: Expression, Structure, Function, and Role in Vacuole Escape

**DOI:** 10.3390/microorganisms8121933

**Published:** 2020-12-05

**Authors:** Waad Bajunaid, Nathaline Haidar-Ahmad, Anwer Hasil Kottarampatel, France Ourida Manigat, Navoun Silué, Caetanie F. Tchagang, Kyle Tomaro, François-Xavier Campbell-Valois

**Affiliations:** 1 Department of Chemistry and Biomolecular Sciences, University of Ottawa, Ottawa, ON K1N 6N5, Canada; wbaju020@uottawa.ca (W.B.); nhaid010@uottawa.ca (N.H.-A.); akottara@uottawa.ca (A.H.K.); fmani073@uottawa.ca (F.O.M.); nsilu022@uottawa.ca (N.S.); ctcha103@uottawa.ca (C.F.T.); ktoma010@uottawa.ca (K.T.); 2 Department of Biochemistry, Microbiology and Immunology, University of Ottawa, Ottawa, ON K1N 6N5, Canada

**Keywords:** *Shigella*, type III secretion system (T3SS), injectisome, secretion, transcription regulation, virulence, genetically encoded reporter, vacuole rupture, autophagy

## Abstract

*Shigella* spp. are one of the leading causes of infectious diarrheal diseases. They are *Escherichia coli* pathovars that are characterized by the harboring of a large plasmid that encodes most virulence genes, including a type III secretion system (T3SS). The archetypal element of the T3SS is the injectisome, a syringe-like nanomachine composed of approximately 20 proteins, spanning both bacterial membranes and the cell wall, and topped with a needle. Upon contact of the tip of the needle with the plasma membrane, the injectisome secretes its protein substrates into host cells. Some of these substrates act as translocators or effectors whose functions are key to the invasion of the cytosol and the cell-to-cell spread characterizing the lifestyle of *Shigella* spp. Here, we review the structure, assembly, function, and methods to measure the activity of the injectisome with a focus on *Shigella*, but complemented with data from other T3SS if required. We also present the regulatory cascade that controls the expression of T3SS genes in *Shigella*. Finally, we describe the function of translocators and effectors during cell-to-cell spread, particularly during escape from the vacuole, a key element of *Shigella*’s pathogenesis that has yet to reveal all of its secrets.

## 1. Introduction

*Shigella* spp. are important human pathogens infecting the large intestine and responsible for hundreds of millions of infections every year [1,2]. They mainly differ from commensal *Escherichia coli* by the presence of a large virulence plasmid (VP) that encodes the various components of the *Shigella* type III secretion system (T3SS). This comprises the various proteins required to assemble the syringe-like type III secretion apparatus (T3SA), also known as the injectisome, as well as those acting as transcription regulators, substrates, and their cognate chaperones. Some of the T3SA substrates are effectors that rewire human cell biochemical processes through their enzymatic or binding activities. The effectors allow the invasion of the cytosol of epithelial cells by *Shigella*. This entry process is akin to phagocytosis. *Shigella* then uses actin-based motility and effectors to invade the cytosol of neighboring cells in a process named cell-to-cell spread that shares some similarities with the entry [3]. Herein, we discuss the structure and function of the T3SA, the regulation of the expression of T3SS genes, and recent progress in our understanding of the role of T3SA effectors in vacuole escape.

## 2. The Structure of the T3SA

### 2.1. Overview of the T3SA

The T3SA weighs 3.5 MDa and is prevalent among pathogenic proteobacteria; its genetic organization, protein components, and structure are well conserved [4,5]. The structure of the T3SA has been reviewed from a historical and global perspective encompassing data from many bacteria [5,6,7]. Here, we focus on the most recent advances about the structure of the T3SA of *Shigella*. In some cases, though, data from other bacterial systems are used to complement this model. We will refer generically to the various proteins forming the T3SA as its parts and use their *Shigella* names throughout, but at the first occurrence where their name in the unified nomenclature is mentioned [5]. *Shigella* harbors a ~232 kbp virulence plasmid (VP) that is maintained by an intricate combination of toxin–antitoxin systems [8] and harbors most T3SS genes [9]. The parts of the T3SA in *Shigella*, as in other species [4], are encoded on a single DNA fragment of approximately 30 kbp named the entry region and composed of the *mxi/spa* and *ipa* operons [10,11]. In permissive conditions (e.g., temperature > 32 °C), the *mxi/spa* and *ipa* operons produce approximately 20 different protein parts that yield the T3SA once assembled together. This nanomachine is composed of three segments: the extracellular needle and the tip complex that protrudes on the surface of the bacteria, the transmembrane basal body spanning the outer and the inner membranes as well as the cell wall, and the cytosolic complex, also known as the sorting platform. When the T3SA is active, the protein substrates are selected and unfolded by the sorting platform. Then, substrates through the action of the export apparatus successively transit through a narrow secretion channel in the center of the basal body and the needle, and a pore of similar width formed by the insertion of a translocon into the host plasma membrane [12]. At the end of their journey, the substrates are finally delivered in the host cytosol where they can refold to their native state.

### 2.2. The Detailed Structure of the T3SA

**Figure 1:**
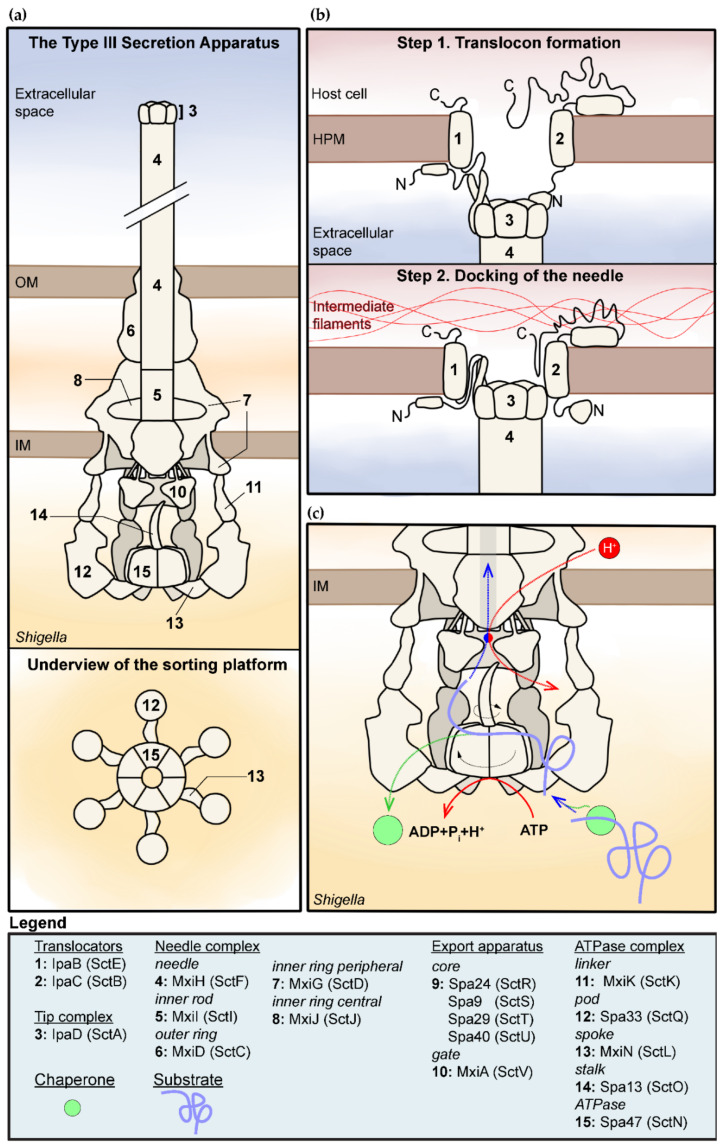
Structure and function of the type III secretion apparatus (T3SA) in *Shigella* spp. (**a**) Overview of the structure of the inactive T3SA. Note that the tip complex and cytosolic components of the T3SA are represented with a 3D perspective while the body is represented as a flat longitudinal cross section [16,17,31,32]. The bottom panel represents the sorting platform viewed from the cytosol of the bacterium. (i.e., viewed from the underside). (**b**) Model for the formation of the translocon and mutual interaction with the tip complex, the host plasma membrane, and the intermediate filaments [33,34]. (**c**) This is a model for the secretion of T3SA substrates summarizing elements discussed in the text. It indicates the role of the chaperone and ATPase–stalk complex in the unfolding of the substrates (purple) through rotation induced by ATP hydrolysis [35,36], and of the gate of the export apparatus in the creation of a proton motive force required for substrates secretion [37,38]. Movement is represented by dashed arrows; rotation is represented by curved arrows. The coupling of the protein substrates–proton antiporter is represented by the red–blue circle. The legend indicates the name of the various components in *Shigella* and in the unified nomenclature in parentheses.

Structures of the needle complex composed of the basal body and the extracellular needle are known since the turn of the 21st century [13,14]. Since then, near-atomic details of several parts of its structure have been described [5,15]. The structure of the intact membrane-embedded T3SA was elusive until two seminal studies provided a first glimpse of it [16,17]. These studies and others described below delineate the knowns and unknowns of the structure of the T3SA (Figure 1a). Unless indicated otherwise, the structural models described in this section represent the inactive state of the T3SA.

Compared to the flagellum, its distant relative, the most distinctive part of the T3SA is arguably the extracellular needle [18]. The needle protrudes 50 nm from the outer membrane and provides a 1.5–2.5 nm wide secretion channel [19]. The needle has a helical symmetry resulting from the homopolymerization of approximately 100 MxiH (SctF) subunits, which adopt an α-hairpin structure [20,21,22]. The needle is capped with the tip complex that adopts an elongated shape slightly wider than the needle, giving the ensemble a scepter shape. The tip complex in the inactive state has been proposed to be made of an homopentamer of IpaD or an heteropentamer comprising four IpaD and one IpaB [23,24]. The source of discrepancy between the two models could be due to the experimental conditions, as others found that IpaB is more readily detectable at the tip of the needle upon exposure to bile salts, which bind IpaD and prime the T3SA for activation [25,26]. This IpaB–IpaD heteropentamer may thus represent a configuration of the tip complex that is intermediate between those encountered in the inactive and the active state of the T3SA [27]. Interestingly, genetic data indicate that IpaD and IpaB are both essential to block the constitutive secretion of substrates [3,28], suggesting that IpaB, particularly its N-terminus [29,30], is contributing to the stabilization of the tip complex in a closed conformation (Figure 1a).

The frame of the basal body is composed of an outer ring (OR) with mixed C15–16 cyclic symmetry standing on the inner rings (IR) with C24 cyclic symmetry [39]. The OR is composed of the secretin MxiD (SctC), which extends into the periplasm where it contacts the peripheral IR composed of MxiG (SctD). The central IR composed of MxiJ (SctJ) sits under the OR and is circled by the peripheral ring [39] (Figure 1a). The needle is inserted in the basal body through interactions with the inner rod, also known as the needle adapter [40], which is composed of one or two helical turns of homopolymerized MxiI (SctI). Cryo-EM revealed the stoichiometry of the export apparatus; it is composed of five Spa24 (SctR), four Spa9 (SctS), and one Spa29 (SctT) [41]. The core complex of the export apparatus has an inverted conical shape [41] and is located right beneath the inner rod within the periplasmic region of the OR and the IR [17,39,42]. The apex of the core is embedded in an area of the cytosolic membrane sitting below the IR and that appears highly curved and thinned in its vicinity [32]. Based on the flagellum, a single copy of Spa40 (SctU) would be located near the apex of the core [43]. By contrast, the gate of the export apparatus named MxiA (SctV) was detectable only in intact T3SA [16,17]. The insertion of the N-terminus of MxiA in the cytosolic membrane positions its nonameric C-terminus domain [44] in the cytosol at the entrance of the secretion channel, just beneath the core of the export apparatus [16,17,32].

Cryo-ET indicated that the sorting platform sits just below the MxiA ring (Figure 1a) [16]. The proteins forming the export apparatus and sorting platform were located in the intact T3SA tomograms by measuring the loss of electrons absorbance in mutant strains or the appearance of new electron-dense areas when complementing a relevant mutant with the corresponding Green Fluorescent Protein (GFP) fusion [16,17]. These data indicated that the sorting platform is arranged in a six-fold symmetry structure reminiscent of a pinwheel (Figure 1a). It is characterized by a central hub density with six spokes harboring a pod at their periphery. This pod-like structure forms a “cage” around the gate of the export apparatus. This structure differs significantly from the corresponding C-ring “wall” of the flagellum [16,45,46]. The hub of the sorting platform is formed by the stalk Spa13 (SctO) inserted in the central pore of the homohexameric ATPase Spa47 (SctN) [16,36,47]. From this ATPase complex, six MxiN (SctL) spoke-like linkers radiate outward and are each attached at their edge to a Spa33 (SctQ) pod. Each of these is attached to one MxiK (SctK), which directly binds the cytosolic domain of the peripheral IR formed by MxiG [31]. Spa33 may also form a heterodimer with a short version of itself obtained through an alternative translation site; both versions of Spa33 are required for the assembly of functional T3SA [48]. Nonetheless, it is not clear at the moment where this heterodimer, let alone more complex oligomers [49], might be located within the pod [16].

### 2.3. The Assembly of the T3SA and the Orderly Secretion of Its Substrates

The assembly of the basal body of the T3SA has been studied in several bacteria, yielding two alternative models: the inside-out and the outside-in model [5,7,15]. The first step is sec-dependent but unique to each model, while the second step is T3SA-dependent and common to the two models. The first step of the inside-out model consists in the nucleation of the IR around the core components of the export apparatus already embedded in the inner membrane [50,51,52]. Then, the OR would assemble, helped or not by a pilotin [53], and join the IR to form the basal body. This model is favored for the flagellum and the injectisomes of *Salmonella* (SPI-1 T3SA) and *E. coli* (enteropathogenic and enterohemorrhagic) [5]. The assembly of the T3SA has not been thoroughly characterized in *Shigella* [41,54,55], but the similarity with the SPI-1 T3SA suggests that it should follow the inside-out model. By contrast, in *Yersinia*, the assembly of the T3SA was instead hypothesized to proceed from the outside in. The key difference with the first model is that the secretin is required to stabilize or allow the insertion of the IR in the cytosolic membrane [56]. Further studies are warranted to demonstrate whether T3SA assembly indeed varies between species.

**Figure 2:**
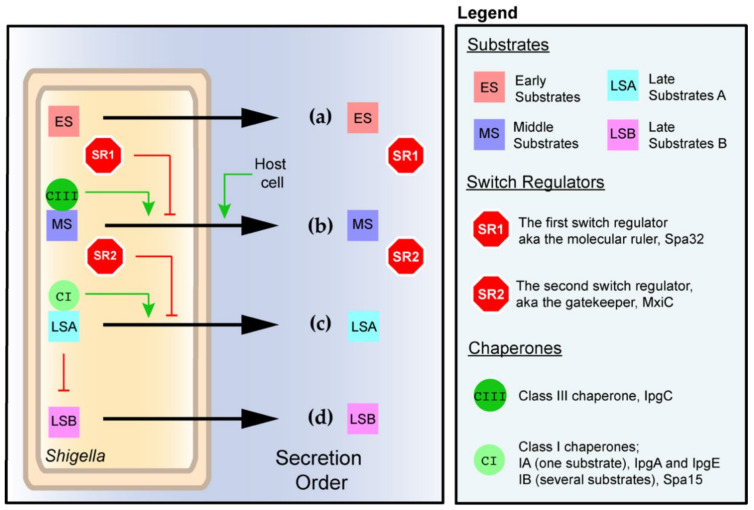
The ordered secretion of the T3SA in *Shigella* and its main regulators. (**a**) The early substrates are secreted as soon as expressed to assemble the inner rod and needle. The concomitant secretion of the first switch regulator Spa32 allows secretion of small amounts of middle substrates IpaD and IpaB to yield the inactive T3SA represented in Figure 1a. (**b**) The contact with host cells activates the T3SA, allowing the secretion of middle substrates and the second switch regulator MxiC. This, in turn, triggers the successive secretion of late substrates A (**c**) and late substrates B (**d**). The black arrow represents the secretion by the T3SA. The control of the production of late substrates B by late substrates A is described in Figure 3.

In both models, the recruitment of the sorting platform to the basal body is critical to obtaining secretion-competent T3SA. Indeed, in the absence of the sorting platform, the secretion channel in the basal body appears close, putatively preventing premature secretion [16,17]. Of particular importance at this stage are the early substrates Mxi and MxiH, which upon secretion spontaneously homopolymerize to form the inner rod and needle. Then, a small fraction of the cytosolic store of the middle substrates IpaB and IpaD is secreted to form the tip complex at the top of the needle. This step yields the fully functional but inactive T3SA. Despite his name, this state is indeed characterized by a leaky secretion, which corresponds to the release of only a small quantity of the middle and late substrates [57]. Finally, T3SAs switch to their active state upon contact with host cells or treatment with the chemical inducer Congo red [28,57]. This triggers the ordered secretion of the middle substrates followed by that of the late substrates. In *Shigella*, the late substrates were characterized as either first wave or second wave effectors [3], which we propose naming late substrates A and B for the sake of simplicity. The VP-encoded secretome of the T3SA in *Shigella flexneri* str. M90T consists of 35 protein substrates that were confirmed or identified for the first time by mass spectrometry analyses of the secretome [58]. Most of these substrates are encoded by T3SS-related genes. The exception resides in four antitoxins from type II toxin–antitoxin systems, which are minor late substrates that warrant further study [58]. Below, we describe how the T3SA parts and accessory proteinaceous factors regulate the ordered secretion of the T3SA substrates (Figure 2).

As hinted above, MxiI and MxiH are the early T3SA substrates and form the inner rod and the needle (Figure 2a). Their secretion necessitates the association of the sorting platform with the basal body [16,17,31] and the ATPase activity of Spa47 as described below. The length of the needle is controlled by the first switch regulator dubbed the “molecular ruler,” which is homologous to that originally characterized in *Yersinia* [5,59]. Indeed, the absence of the homologous *Shigella* protein Spa32 (SctP) confers extra long needles and poor secretion of middle substrates [60,61,62]. The detailed mechanism through which Spa32 measures needle length is still debated [5]. Nonetheless, Spa32 interacts with the core export apparatus component Spa40 and suppresses its autocleavage [62,63], thereby favoring the secretion of the early substrates. Concomitantly with the needle reaching the proper length, Spa32 is secreted [60]. This event hence relieves the inhibition of the autocleavage of Spa40 [64]. This cleavage is hypothesized to modulate the interactions that Spa40 may form with other components or regulators of the T3SA, rather than its tertiary structure [65], thereby switching the secretion from early to middle substrates [62] (Figure 2b).

The critical event that activates T3SAs is the sensing of host cells by the tip complex. In response to this event, the tip complex undergoes a first conformational change leading to the exposure of IpaB at the tip. From this position, IpaB can then insert its C-terminus transmembrane domain in the host plasma membrane (HPM) [25,27,30]. This second conformational change leads to the secretion of IpaC, which the C-terminus inserts in the HPM as well, allowing the formation of the translocon through the formation of a transmembranar heteromer with IpaB [30,66]. The stoichiometry of IpaB and IpaC in the translocon, however, is still unknown [67]. The translocon width in *Salmonella* is approximately 13.5 nm [68], and the diameter of its pore at about 2.5 nm is comparable to that of the secretion channel [69]. The translocon seems to induce a tent-like distortion of the host PM around it [68]. This might be due to the cytoskeleton-manipulating function of IpaC. Indeed, IpaC is known to hijack the actin microfilaments during host cell invasion [70]. It was also found to bind vimentin intermediate filaments, a property shared with orthologs in other T3SS [33]. The binding of the C-terminus of IpaC to vimentin is instrumental to inducing a conformational change that extends from the binding interface to the extracellular N-terminus of IpaC [34,71]. This was hypothesized to permit the docking of the tip complex to the translocon (Figure 1b). Interestingly, the tip complex associated to the translocon appears narrower than the needle [68], contrary to what was reported in the inactive state [23,24]. This is perhaps a consequence of the conformational changes induced by the docking of the tip complex into the HPM translocon.

Let us take a step back and discuss how the conformational change at the tip complex is signaled down to the export apparatus and the sorting platform. The identification of IpaB and IpaD mutants that assemble a functional tip complex in the inactive state (e.g., capable of blocking constitutive secretion), but deficient in the active state (e.g., incapable of host cell invasion or Congo red induction), suggests that both proteins play a direct role in the signaling of secretion activation [30,66,72]. This message is probably signaled toward the basal body through the needle since some MxiH mutants are capable of forming normal needles but neither respond to Congo red nor invade cells [73]. Presumably, the message is then communicated to the gate of the export apparatus, the sorting platform, or both through an unknown mechanism likely involving the inner rod, the core of the export apparatus, and probably the peripheral IR [32].

The key player of the sorting platform is the ATPase Spa47. As several ATPases, Spa47 is a homohexamer in its holo form [16,36,47,74,75]. This quaternary structure is stabilized by MxiN, which reduces futile ATP hydrolysis prior to the association of the sorting platform with the needle complex [76,77]. The main role of the ATPase is to strip the chaperone of its substrate and unfold the latter to render it secretion-competent [78]. Spa47 is helped in this and in the coupling with MxiA by the stalk Spa13 [79]. A chaperone was suggested to directly interact with the surface of the ATPase located within the export gate cage of the injectisome of *Salmonella* [35]. As yet, this model has not been confirmed in *Shigella*. A recent structure of the complex formed by the Spa47–Spa13 homologs in enteropathogenic *E. coli* suggests the rotation of the ATPase and the tilt of the stalk may couple substrate unfolding to the activation of the gate of the export apparatus MxiA [36]. Intriguingly, the rotation of the ATPase complex may be responsible for the needle-complex-induced rotation observed in living cells of *Pseudomonas aeruginosa* [80,81].

On the other hand, the threading of substrates through the ATPase complex pore is unlikely because this site is occupied by the stalk [16,36,47]. Taken together, these findings support the notion that the substrates, through their cognate chaperone, their secretion signal, or both, are recruited directly on the surface of the ATPase complex that is located within the export gate cage in intact T3SAs. By contrast, it has been proposed that substrates may also be recruited through cytosolic pools of Spa33–MxiN–Spa47–chaperone–substrate or Spa13–chaperone–substrate complexes [7,79]. In the case of the flagellum, a tripartite complex between a substrate and its chaperone and the export gate was reported [82], suggesting the gate might also act as a docking site for substrates. This model, however, has not been validated for the injectisome of *Shigella*; it is also difficult to reconcile with the substrate unfolding role of the ATPase. The hydrolysis of ATP, the electrostatic repulsion between the substrates and the secretion channel, and the potential energy of the unfolded polypeptides have been suggested to participate in powering secretion, but the proton motive force (PMF) is currently considered the most important contributor [38,83]. The flagellum export gate was suggested to act as a proton–protein antiporter [37], thus enabling the secretion of substrates (Figure 1c). Whether the export gate of injectisomes play a similar role or not is unknown. It is noteworthy that ATP hydrolysis should increase the local proton concentration in the vicinity of the export gate, but the role of this in secretion is unclear since it seems it would disfavor antiport secretion by dissipating the local proton gradient.

The selection of middle substrates and late substrates among thousands of cytosolic proteins depends on the presence of a degenerate polar and disordered 20 amino acids N-terminal signal that is not cleaved during processing [5]. The secretion of middle substrates is prioritized over the late substrates through autochaperoning activity in the case of IpaD or binding to the chaperone IpgC in the case of IpaBC, and the action of the second switch regulator MxiC (SctW) [84,85], which is also known as the gatekeeper. At this stage, cytosolic MxiC inhibits the secretion of late substrates, hence favoring the secretion of the middle substrates [84,85] (Figure 2b). It was hypothesized to do so by binding to the inner rod [86,87]. MxiC appears to be a promiscuous binder since interactions with Spa47, MxiA, and Spa33 were also reported [49,86,88]. For example, MxiA mutants preventing MxiC binding partly disrupted the secretion hierarchy of substrates [88]. Nevertheless, how these interactions between MxiC and different parts of the T3SA are orchestrated to permit substrate class switching is unclear.

Mutation to alanine of a conserved patch of charge residues lining the lumen of the needle inhibits secretion in several species, suggesting a role for the needle in the transport of substrates [19]. It is unknown, however, whether unfolded substrates, once engaged, can backtrack or if rather they are poised to move toward the extracellular medium due to the properties of the needle, as electron micrographs of T3SA trapped with a GFP cargo seemed to indicate [89]. When secretion can proceed unimpeded, though, the cytosolic store of the middle substrates is eventually exhausted [90]. It is noteworthy that the cytosolic store of IpaBCD greatly exceeds the amount required for the formation of the tip complex and translocon. This ensures the tight repression of secretion in the inactive state; once T3SAs are activated, however, the cytosolic store of translocators must be significantly depleted, if not exhausted, before the secretion of late substrates is finally permitted (Figure 2c).

Concurrently with the exhaustion of the middle substrate store, MxiC is secreted [84,85]. The needle was suggested to play a critical signaling role in the trigger of the secretion of MxiC [91]. The depletion of the cytosolic store of MxiC allows MxiA to secrete the late substrates [88]. The late substrates A, which are stored in the cytosol prior to T3SA activation, possess a secretion signal and their own chaperones, which belong to a different structural class than their translocator-binding counterpart [6]. Hence, it was proposed that the structural diversity of chaperones contributes to establishing the secretion hierarchy through their binding with varying affinity to the ATPase [92]. This phenomenon may also be modulated by MxiC. By contrast, the late substrates B, which are only produced following the secretion of late substrates A (Figure 2d and next section), are unchaperoned and secreted as soon as produced [93]. This suggests that the secretion signal suffices for the secretion of late substrate B and that their transcription serves merely as another manner of hierarchizing secretion further.

## 3. The Regulation of the Expression of the T3SS in *Shigella*

### 3.1. Overview of the Regulatory Cascade of the T3SS

**Figure 3:**
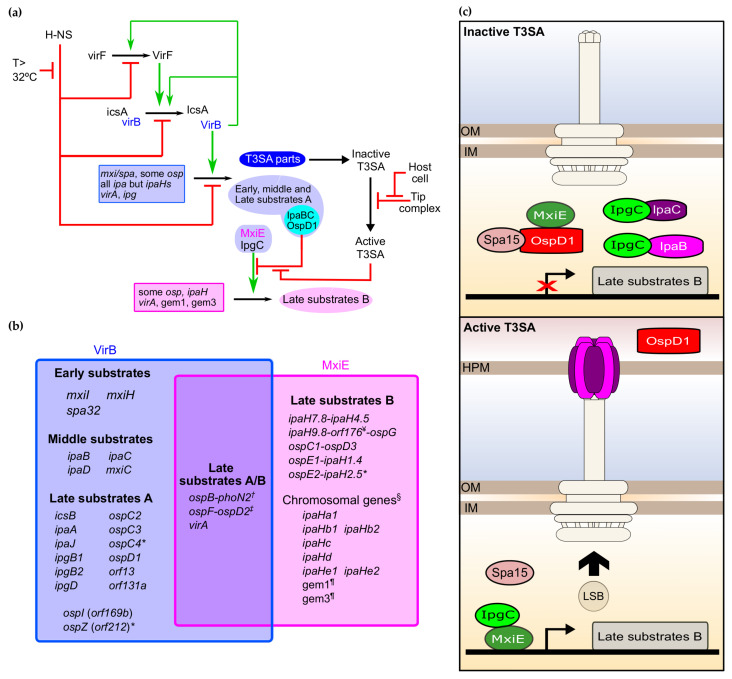
The transcription regulatory cascade of the T3SA. (**a**) Key transcriptional regulatory cascade of the type III secretion system (T3SS) in *Shigella*. (**b**) The VirB and MxiE regulons; this figure is inspired from a prior version [95], and updated to take into account the most recent findings [58,96]. * These are likely pseudogenes in strain M90T; ^†^ this gene belongs to the VirB and MxiE regulon but its product is not a T3SA substrate; ^‡^ this gene is weakly expressed and a new addition to the MxiE regulon; ^§^ the chromosomal *ipaH*s are annotated according to [97]; ^¶^ whether the product of these genes are T3SA substrates is currently unknown; ^¥^ this gene encodes an antitoxin from a TA system that is weakly secreted by the T3SA. Note that other antitoxin coding genes *gmvA* (orf48), orf86, and *mvpA*, which are not part of these two regulons, were also suggested to be T3SA substrates [58]. (**c**) Heteromers formed by MxiE and IpgC in the inactive and active state of the T3SA and their effect on the transcription of late substrates B.

The regulation of the production of proteins encoded by T3SS genes intervenes mostly at the transcriptional level. The T3SS regulatory cascade is composed of a repressor named the histone-like nucleoid structure protein (H-NS), and of three transcription activators named VirF (AraC-family), VirB (ParB family), and MxiE (AraC family) (Figure 3a) [94]. The first three players coordinate the transcriptional response to the rise of temperature occurring upon *Shigella’s* transition from an external environment to its host. The interaction between these factors and DNA culminates with the production of VirB, which activates the expression of most T3SS genes, hence ensuring that the T3SA is assembled in the *Shigella* membrane only when it is inside its host. The last transcription activator MxiE senses the presence of host cells using T3SA activity as a proxy. Indeed, MxiE is inhibited at the posttranslational level when T3SAs are inactive. This inhibition is released when T3SAs are active, allowing MxiE to upregulate the expression of genes encoding late substrates B. Finally, a handful of genes called late substrates A/B are dually controlled by VirB- and MxiE-dependent promoters [95,96] (Figure 3b). Below, we detail the action of these different transcription factors in the regulation of the main virulence genes.

### 3.2. Master Regulators of the T3SS in Shigella: H-NS, VirF, and VirB

The repressor of the T3SS regulatory cascade in *Shigella* is the histone-like nucleoid structure protein (H-NS). It forms homomers in isolation, but may also heteromerize with two related proteins named StpA (prevalent in *Shigella* spp.) and Sfh (*S. flexneri* str. 2457T) [98,99]. Interestingly, StpA and Sfh can complement the loss of H-NS by, for example, ensuring the repression of T3SS genes, thus suggesting that these three paralogs share this function [99,100]. H-NS binds AT-rich DNA through the insertion of a conserved three-residue motif into the minor groove according to molecular dynamics simulations [101]. In response to changing physicochemical conditions, H-NS can toggle between a stiffening or a bridging DNA binding mode [102]. In the filament mode, several H-NS protomers are associated to a single DNA segment, while in the stiffening mode, they bundle two or more DNA segments together [103]. Both binding modes inhibit transcription initiation by impeding the access of transcriptional regulators and the RNA polymerase to promoters. The binding of H-NS to DNA can also hinder transcription elongation, which can favor Rho-dependent termination [104]. To our knowledge, this phenomenon, however, has not been assessed for *Shigella* T3SS genes.

In *Shigella*, T3SS genes have 60% to 70% AT content [10]. H-NS thus has a countless number of potential binding sites spread over the VP. Those located in the entry region have been particularly scrutinized due to their key role in the regulation of virulence. Below 32 °C, H-NS binding is favored, thus imposing three locks on the regulatory cascade of *Shigella*. The two most upstream result in the silencing of *virF* and *virB* that encode two transcription activators; the third is downstream and results in the silencing of T3SA parts and several substrates [99,105,106,107,108]. The main consequence of the switch to human body temperature is to open these three locks. For example, the conformation of the promoter of *virF* favors H-NS binding and silencing of *virF* below 32 °C [107,108]. By contrast, at the human body temperature (or >32 °C), the conformation of the promoter precludes H-NS binding, hence favoring transcription initiation and, ultimately, the production of the VirF protein [107,108]. *virF* integrates other environmental cues to ensure that the virulence genes are expressed to optimal level when the right conditions are met. For example, *virF* is silenced at acidic pH due to the inactivation of the CpxAR two-component system. In neutral conditions, CpxA-activated CpxR upregulates the expression of *virF*. The translation factor EF-P and its regulator PoxA are required for the production of CpxA, and thus play an indirect role in the expression of *virF* [109]. The antisilencing of *virF* is also favored by nucleoid-associated proteins such as IHF and FIS whose functions are modulated by the physiological state of the cell, most notably in function of the growth phase [108,109,110]. Furthermore, the production and the function of VirF are respectively regulated at the translational level through posttranscriptional modifications of several tRNAs [108,111,112], and by the production of an N-terminally truncated isoform of VirF, which, in contrast to its full-length counterpart, represses target genes [113]. As the most upstream transcription activator in the virulence cascade, VirF activates the transcription of *icsA*, which encodes a virulence factor essential for cell-to-cell spread, as well as of *virB*, which encodes the second transcription activator [108]. VirF is a promiscuous DNA binder that tolerates degeneracy within its AT-rich consensus binding site. This property, combined with the high AT-content of T3SS genes, allows the formation of large VirF oligomers bridging discrete binding sites within the same gene [114]. Interestingly, VirF may be acting through the displacement of H-NS around the +1 of transcription and in the coding sequence of *icsA* [114], while in the case of *virB*, it more classically binds to the promoter [105,108]. On top of this antisilencing effect on transcription, VirF was recently suggested to regulate the expression of *icsA* by directly binding its mRNA and its noncoding antisense regulator RnaG [115].

VirB is the key to activating the transcription of T3SS-related genes for which the repression of H-NS function by temperature (>32 °C) is not sufficient. The DNA binding site of VirB is disputed, as independent studies indicated that an eight-nucleotide motif or two inverted repeats thereof separated by a single nucleotide were required for VirB activity [116,117,118]. As suggested in the most recent study, the number of sites matching to the larger recognition site is commensurate with the number of genes controlled by VirB, thus arguing in favor of its relevance. Alternately, noncontiguous eight-nucleotide motifs, provided they can be brought together in space, might act as nucleation sites for the formation of VirB–DNA complexes. VirB is deemed an antisilencing factor because its capacity to control the expression of its target genes depends on the silencing activity of H-NS on them [99,119,120]. Furthermore, VirB binding motifs both upstream and downstream, and sometimes at a considerable distance from the +1 of transcription of several T3SS genes, are documented, suggesting that the formation of large VirB complexes nucleating from these sites may help displace H-NS complexes [106,118,119,120,121,122,123]. The existence of two positive feedback loops of VirB on *virB* and *virF* was proposed to explain the great change of expression of T3SS genes observed upon shifting to the permissive temperature [117] (Figure 3a). The VirB regulon was determined with DNA microarrays by comparing mRNA abundance in Δ*virB* or WT grown at 30 °C versus WT grown at 37 °C. This study showed that T3SS genes are regulated by VirB, thus supporting its role in mediating the effect of temperature on virulence [124]. Although this study was not pangenomic, it probed almost all T3SS genes on the VP. Based on these findings and the mode of action described above, VirB is hypothesized to directly activate the transcription of T3SA parts, early substrates, middle substrates, and late substrates A (Figure 3a,b). Besides transcription regulation, a few cases of posttranscriptional regulation of the virulence regulatory cascade have been reported. First, the mRNA-binding protein and posttranscriptional regulator CsrA acts as a positive regulator of *virF* and *virB* through an unknown mechanism involving the glycolytic pathway [125]. Interestingly, CsrA controls the expression of T3SS genes in other species [126]. In enteropathogenic *E. coli*, the function of CsrA is even coupled to the activity of the T3SAs [127]. Second, VirB was identified in the phosphotyrosine proteome of *Shigella*; a phosphomimetic mutation at one of the two phosphorylated sites phenocopied *ΔvirB* [128]. Further work is needed to identify the putative tyrosine kinase responsible for this posttranslational modification and determine its relevance for pathogenesis.

The fumarate and nitrate reduction factor (FNR), which is a transcriptional activator important for bacterial adaptation to anaerobic conditions [129], is also known to act as a transcriptional repressor through hindering of RNA polymerase recruitment. It usually binds to DNA as a homodimer, which is destabilized strongly by dioxygen [129]. Indeed, in hypoxia, FNR directly represses the expression of *spa32* and *spa33* by binding to their promoter [130]. This led to the formation of partly functional T3SAs characterized by longer needles and poor secretion of middle and late substrates. A RNA-Seq study further described the role of FNR for the adaptation of *Shigella* to anaerobic conditions; the most salient finding was that the absence of oxygen induced an indirect FNR-dependent increase in H-NS repression that contributed to silencing *virF*, *virB*, and their target genes (Figure 3a) [131], which reinforces the notion that *Shigella’s* virulence is stunted in anaerobic conditions. In brief, the control of the key transcription activator VirB by VirF, the presence of positive feedback loops involving VirB, and the three locks imposed by H-NS on the two antisilencers and T3SS genes ensures a 5–10-fold change in the expression level of mxi/spa and *ipaBCD* operons at 37 °C compared to 30 °C [124]. These mechanisms, by ensuring that the assembly of the T3SAs takes place only in the host, allow the optimal use of cellular resources.

### 3.3. The MxiE Regulon

VirB also directly controls the transcription of *mxiE*, which encodes a transcription activator of the AraC family [124] (Figure 3a). The full-length transcript of *mxiE* is the result of transcriptional slippage [132]; this phenomenon might be used in so far unknown environmental conditions to reduce the production of MxiE. Besides, VirB controls the expression of genes *ipgC*, *ipaB*, *ipaC*, *ospD1*, and *spa15* [124], which encode MxiE posttranslational regulators. Hence, VirB indirectly controls the expression of late substrates B through the regulation of these genes and others encoding T3SA parts. When T3SAs are inactive, MxiE and IpgC are segregated by the antiactivator OspD1, and its cognate cargos IpaB and IpaC, respectively. The chaperone of OspD1 named Spa15 also acts as co-antiactivator probably through the stabilization of OspD1 in a conformation that favors binding to MxiE (Figure 3c) [133,134,135,136]. By contrast, when T3SAs are active, IpaB, IpaC, and OspD1 are secreted, allowing the cytosol-resident MxiE and IpgC to oligomerize and activate the transcription of late substrates B (Figure 3c). Such direct coupling of transcription to the secretion activity is as elegant as it is rare. The combination of activator and antiactivator factors affords a switch-like behavior displaying 5- to 50-fold upregulation in expression of the MxiE-regulated genes in the active state compared to the inactive state [90,96,97].

The coelution of MxiE and IpgC from affinity columns supports the existence of an MxiE–IpgC complex [137], but its stoichiometry and relevance in vivo are not known. Furthermore, neither MxiE nor IpgC alone can activate the transcription of target genes, supporting the requirement of a MxiE–IpgC heteromer for transcription activation [135]. All monocistronic genes or short operons regulated by the MxiE–IpgC harbor a 17-nucleotide consensus MxiE box in their promoter [96,97,138]. The conservation of key nucleotides of this box is critical for transcriptional activity [97], most likely because of their role in stabilizing a tripartite promoter–MxiE–IpgC complex. The MxiE box being positioned at −35, MxiE–IpgC is thought to act as an RNA polymerase recruiting factor as expected from classical AraC-like transcriptional activators [138].

Flow cytometry, a DNA microarray study previously mentioned, and a more recent RNA-Seq analysis defined the MxiE regulon, which is composed of eight operons encoding fifteen proteins on the VP and nine chromosomal genes [96,124,134] (Figure 3b). Seven chromosomal genes of this regulon belonged to the *ipaH* family and likely originated from the duplication of one or more ancestral *ipaH* genes from the VP [96,124]. The pangenomic RNA-Seq study added two previously unknown chromosomal genes temporarily named gem1 and gem3 that have no homology with T3SS genes to the MxiE regulon [96]. Further studies are required to determine whether they encode proteins that are T3SA substrates.

## 4. Genetically-Encoded Reporters to Monitor the Activity of the T3SA

### 4.1. Transcription-Based Assays

**Figure 4:**
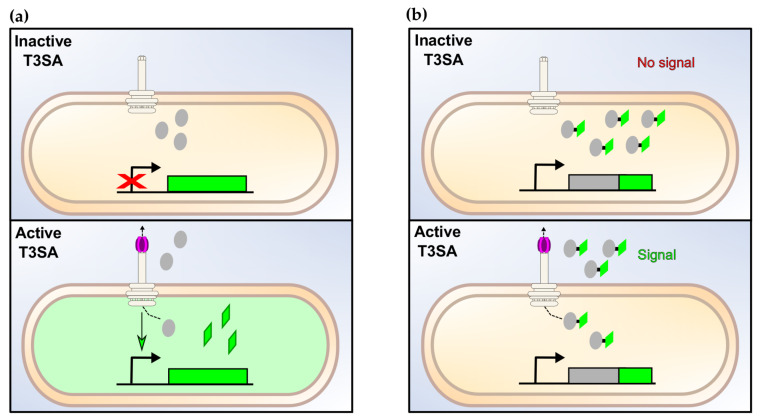
The types of assays to measure the activity of the T3SA. (**a**) Transcription-based assays. The activation of T3SAs upregulate the expression of the reporter gene, thus leading to an increased production of the corresponding assay proteins. The relative secretion activity is then estimated by the measurement of the fluorescence or of any other relevant signal emitted by the assay protein (e.g., luminescence, colorimetric, etc.). (**b**) Secretion-based assay. Upon activation of the T3SA, T3SA substrates fused to a specific tag are secreted. The relative amount and location of the substrates inside host cells or in the extracellular medium are measured with the relevant method, as described in (**a**).

Bacterial adaptation occurs mainly through transcriptional regulation as illustrated in the virulence regulatory cascade of *Shigella*. Hence, transcriptional reporters using relevant promoters and reporter proteins such as the GFP (Figure 4a) can be easily designed to monitor critical elements of the bacterial physiology including T3SAs activity [139]. In *Shigella* spp., the regulation of late substrates B through their MxiE box provided a simple solution to develop a transcription-based secretion activity reporter (TSAR). The TSAR’s key feature is to put the production of a fast-maturing variant of GFP under the control of a MxiE-regulated promoter [90]. The TSAR demonstrated that the T3SA is not always active but rather follows cyclic activation and deactivation as it encounters and leaves plasma membrane compartments during the various stages of intracellular life [90]. The TSAR has proved useful to understand other elements of pathogenesis of *Shigella* [33,34,140,141,142,143]. It has also allowed monitoring of the tissue distribution and spatiotemporal parameters of T3SA activity in a guinea pig model of shigellosis [144,145].

Recently, transcriptional reporters have also been used to identify the factors affecting the activity of the T3SA in other bacteria. A fluorescent cytosolic dual-color reporter with a constitutive GFP put under control of the promoter of *uhpT (uhpTp*::GFP*)*, which is induced by glucose-6-phosphate, was used to detect cytosolic *Shigella* or *Salmonella* [146,147]. Recently, an improved dual reporter system with constitutively expressed DsRed and *uhpTp*::sfGFP indicated that strains expressing SopE ruptured their vacuole more rapidly than their counterparts that were devoid thereof [148]. Interestingly, another study provided evidence that the SPI-1 T3SA is active in cytosolic bacteria [149], suggesting the absence of T3SA activity observed in cytosolic *Shigella* is not universal [90]. In *Yersinia*, a reporter system containing an expression cassette composed of *yopHp*::mCherry was used to monitor the effect of T3SS small molecule inhibitors and help define their mechanism of action [150]. A similar reporter based on the *yopE* promoter was used in a *Yersinia* mouse infection model. This reporter displayed maximal activity in bacteria located at the periphery of splenic microcolonies. This study drew attention to the key role of peripheral *Yersinia* in the maintenance of a shelter for their kins in the center of the microcolonies, as they fenced off host immune cells using their T3SAs [151].

### 4.2. Direct Secretion Assays

The applicability of transcriptional reporters is limited by the delay between the event of interest and the accumulation of sufficient reporter proteins for detection. To circumvent this caveat, various secretion assays have been developed to decipher the expression, secretion, translocation, activity, and localization of bacterial T3SS effectors within host cells (Figure 4b). The measurement of the secretion activity by GFP-substrate fusions is precluded by the clogging of T3SAs due to the incapacity of the ATPase SctN to unfold the GFP [78,89,152]. An alternative is the split-GFP in which the eleventh β-strand of the GFP (GFP11) spontaneously associates with the first 10 strands (GFP1-10) [153].

The tagging of T3SA substrates with the short and unfolded GFP11 allowed the recovery of GFP fluorescence upon its secretion in the cytosol of the host cell and association with GFP1-10. Nonetheless, the maturation time of the split-GFP was estimated to two hours [154], thus introducing a large dead time in the measurements. Despite this caveat, the split-GFP system was used to track the location of effectors OspF and OspG in plant cells by confocal microscopy [155]. Alternatives to GFP also include the light–oxygen–voltage domain (LOV) that belongs to the flavin-binding protein family, and the fluorescence-activating and absorption-shifting tag (FAST). By comparison with the full-length GFP, LOV and FAST are smaller, mature faster, and do not inhibit secretion, nor do they require oxygen to fluoresce. A variant of iLOV named phiLOV was used to monitor IpaB production and secretion [156]. Although the real-time imaging of IpaB–phiLOV was not reported, a substrate from another T3SA was tracked successfully with the same technique [156]. The poor brightness of phiLOV and LOV domain may, however, limit their use in live imaging. Similarly, the fusion of FAST to OspF and IpaB allowed to track them within infected host cells [157]. FAST is fluorescent through binding to exogenous small molecules with different fluorescent properties, thus allowing flexible imaging modalities. Similarly, the self-labeling enzymes SNAP-tag, CLIP-tag, and HaloTag work in both aerobic and anaerobic conditions, and can be labeled with different fluorophores. They were used to track T3SS effectors in *Salmonella*, and should be easily adapted to *Shigella* [158].

In place of these large protein fusions, smaller tags that allow direct labeling have been developed to minimize interference with effector secretion and function. A classical example is the tetracysteine motif (4Cys-FlAsH) and its ligand, the fluorescein-based biarsenical dye (FlAsH), which were used to track the translocation of IpaB and IpaC inside human epithelial cells [159]. This method has not been widely adopted, probably due to the toxicity and reaction of the dye with other cell constituents. An emerging alternative is the labeling of protein of interest with fluorescent nonstandard amino acids that are inserted during translation. This method was recently used to image the colonization of the gut by a probiotic *E. coli* strain with high spatial and temporal resolution [160]. The key advance of this study was the continuous in vivo incorporation of a fluorescent unnatural amino acid, which allowed long-term imaging previously impossible. Thus, this approach may prove useful in the future to monitor the secretion and location of T3SA substrates by microscopy.

Classical immunofluorescence approaches were used to monitor the production and localization of many effectors [90,140,161,162], but their low sensitivity can curtail detection of substrates in low amount. By contrast, the SunTag uses a GCN4 antibody–peptide pair to multimerize >10 copies of GFP to a protein of interest, thereby amplifying the fluorescence signal in both living and fixed samples [163]. This method was used to locate IcsB on the *Shigella*-containing vacuole [164], and to demonstrate the colocalization of IpaH9.8 with its substrate hGBP1 [165].

Higher throughput assays have also been developed. For example, in vitro semiautomated solid-plate and liquid assays based on epitope tagging of substrates and Congo red induction of the T3SA was used to measure the role of chaperones in the secretion of effectors [93]. Second, β-lactamase FRET (fluorescence resonance energy transfer) and colorimetric assays, using as reporter substrates the cephalosporins CCF2 and nitrocefin, respectively, confirmed novel T3SA substrates identified by mass spectrometry in a multiwell format [58]. Furthermore, the sensitivity of the FRET assay was increased about 50% with TEM3 M182T, a derivative of TEM1 β-lactamase with higher activity against cephalosporins [166]. Further optimization of the cargo increased the sensitivity of this assay further, thus allowing to more readily detect the T3SA activity against T lymphocytes [166,167], which are readily injected with substrates, but poorly invaded [168]. Surprisingly, the sensitivity of the colorimetric assay with the TEM3 M182T decreased, suggesting that testing additional TEM1 derivatives with improved activity against cephalosporins might be worthwhile to further the versatility and sensitivity of this method. Due to its enzymatic nature, the β-lactamase assay is more sensitive than those with no amplification, but this is at the expense of a loss of spatial information due to the free diffusion of the fluorescent product resulting from CCF2AM cleavage. It is noteworthy that anterior studies about T3SA secretion assays have been reviewed elsewhere [139,169].

## 5. Mechanisms of Vacuole Rupture and Escape in *Shigella*

### 5.1. The Intracellular Niche of Shigella

**Figure 5:**
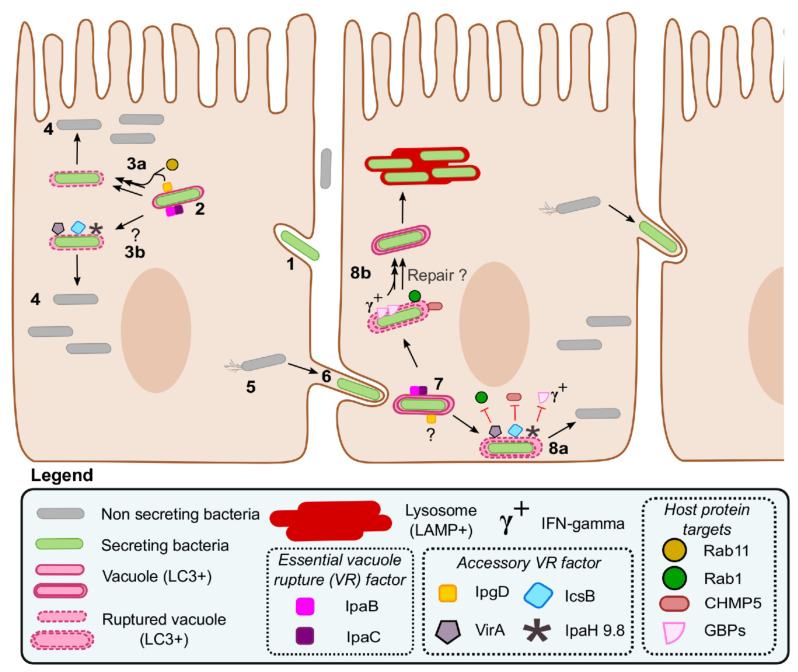
*Shigella* intracellular lifestyle and role of its virulence factors during vacuole escape. The invasion of a nonphagocytic host cell starts by the activation of T3SAs, which induce the remodeling of the plasma membrane through the action of their translocators and effectors (1). The intracellular bacterium is captured in a single membrane entry vacuole; several T3SA substrates, including the translocators IpaB, IpaC, and IpgD, accumulate in the vicinity of the vacuole (2). IpaBC form pores that are essential to vacuole rupture; the IpgD-dependent recruitment of Rab11 might accelerate this process (3a); although the other accessory vacuole rupture factors IcsB, VirA, and IpaH9.8 are dispensable during entry in epithelial cells, they might be important in yet undiscovered conditions such as in immune cells or in specific cytokinic contexts (3b). Cytosolic bacteria proliferate (4) and move using actin comets (5). Upon contact with the plasma membrane, the T3SA is reactivated, allowing the formation of a protrusion (6) and a double membrane dissemination vacuole (7). IpaB and IpaC initiate the rupture of the double membrane dissemination vacuole; the role of IpgD at this stage is unknown. The accessory VR factors IcsB and VirA, and probably IpaH9.8 in the presence of interferon-gamma (IFNγ), facilitate the escape of *Shigella* from the dissemination vacuole already partly ruptured by IpaBC (8a); this is probably realized through the inhibition of their respective targets CHMP5, Rab1, and GBPs. These WT bacteria can then resume cell-to-cell spread. By contrast, in the absence of these accessory VR factors, the repair of the partially ruptured vacuole is induced by Rab1 and CHMP5 (8b), a process that might also be facilitated by the action of the GBPs when IFNγ is present. These mutants (8b) are more often captured in lysosomes and partly deficient in cell-to-cell spread.

*Shigella spp.* enter host cells using an invasion mechanism shared with some bacteria expressing a T3SS such as *Salmonella enterica* [170]. Indeed, both bacteria force their entry into epithelial cells by manipulating the actin cytoskeleton. As in phagocytosis in macrophages, this results in the formation of a bacteria-containing vacuole. While intracellular *S. enterica* adopts a vacuolar lifestyle thereafter, *Shigella* ruptures its vacuole to access the cytosol of its host cell (Figure 5). Then, using IcsA to form actin comets, it initiates cytosolic movement in order to invade neighboring cells [171]. This process named cell-to-cell spread consists of: (1) the formation of a finger-like protrusion by a *Shigella* cell whose movement deforms the plasma membrane of the initially infected host cell and a neighboring cell; (2) the release of this *Shigella* cell into the cytosol of the neighboring host cell through the maturation of the protrusion into a dissemination vacuole and the rupture of the latter.

The formation of the dissemination vacuole and its rupture depends on the T3SS, thus sharing some similarities with the entry. Nevertheless, due to the affixation of the plasma membrane of both host cells, the dissemination vacuole has a double membrane. This might explain the critical role of additional effectors in the escape from the dissemination vacuole, as described below.

### 5.2. Effectors Implicated in the Escape from the Dissemination Vacuole

The rupture of both entry and dissemination vacuoles requires the secretion of translocators IpaB and IpaC [90,172,173,174,175]. Interestingly, the exchange of translocators from vacuole-resident *Salmonella* with those of cytosolic-resident *Shigella* sufficed to swap their final destination [176]. This suggests that the translocators are major players in vacuole rupture that act upstream of most effectors. A noteworthy exception is the PI(4,5)P2 phosphatase, IpgD, whose recruitment of Rab 11 contributes to vacuole rupture [177].

Xenophagy is a form of autophagy constituting a major cell autonomous defense mechanism. Indeed, xenophagy targets intracellular microbes to lysosomal degradation, thus restricting their growth. As in canonical autophagy, ATG8/MAP1LC3 proteins are the gold standard marker of xenophagosomes such as those triggered by the recognition of polyubiquitin chains accumulating on the bacterial membrane or on ruptured vacuole membranes, and on LC3-associated phagosomes [178]. The infection of tissue culture cells yields a small fraction of cytosolic *Salmonella* that is preferentially targeted by xenophagy [179,180,181]. By contrast, *Shigella*, despite LC3 presence on the entry vacuole [140], and the induction of an mTor-dependent amino acid starvation response that is proautophagic [182], is less susceptible than *Salmonella* to xenophagy ensuing from the rupture of the entry vacuole [183,184]. The shorter vacuole residence time of *Shigella* due to its translocators and the cytosolic movement enabling escape from ruptured vacuoles are probably the key factors preventing its capture by the autophagy machinery following entry into epithelial cells.

Nonetheless, the escape of *Shigella* from the dissemination vacuole requires additional effectors such as IpaH9.8, VirA, and IcsB. The first reported example implicated IcsB, whose absence resulted in reduced bacterial fitness and increased labeling with LC3-GFP [185]. This study proposed that IcsB protected IcsA from direct recognition by the autophagy core component ATG5 to prevent the capture of *Shigella* in autophagosomes. To our knowledge, this is the only reported case in which a specific microbial protein is used to initiate xenophagy [178]. Moreover, in the absence of IcsB, *Shigella* is captured in septin cages that restrict its movement [186]. The presence of septin cages around *Shigella* was correlated with that of the ubiquitin-binding proteins SQSTM1 (p62) and NDP52, which are known to favor autophagosome formation. More recently, it was reported that septin cages and LC3 were enriched around dividing bacteria [187]. Furthermore, LC3 recruitment occurred exclusively on vacuole-associated bacteria with active T3SAs, but not in cytosolic bacteria with inactive T3SAs (Figure 5). The amount of LC3 labeling was higher around Δ*icsB* than on the WT solely during cell-to-cell spread [140]. Interestingly, NDP52 was also enriched on secreting bacteria, suggesting that they might be the subpopulation targeted by septin cages. In this regard, it is noteworthy that the division of *Shigella* daughter cells seems coupled with their presence in the dissemination vacuole, perhaps because this step of the cell division is slower in this compartment than in the cytosol [90].

Taken together, these results suggested that LC3 recruitment was occurring by default on *Shigella*-containing vacuoles and that IcsB acted downstream of translocators to favor vacuole escape rather than in blocking xenophagy. In agreement with this notion, time-lapse video microscopy data suggested that the accumulation of LC3 on the dissemination vacuoles formed by Δ*icsB* was the consequence of the failure or retardation of vacuole escape [188]. IcsB was also suggested to play an active role in favoring cell-to-cell spread by recruiting the actin-associated protein TOCA-1 and forming an actin-rich structure, dubbed an actin cocoon, around the *Shigella*-containing vacuole [162,189,190,191]. Recently, compelling biochemical data indicated that IcsB is an 18-carbon fatty acid lysine N^ε^-acyltransferase of at least 60 host proteins, including several SNAREs, septins, and small GTPases [164]. The addition of an IcsB-mediated lipid anchor to the C-terminus of Rho GTPAses was suggested to perturb their function by preventing their normal membrane cycling. Distant homologs of IcsB named the Rho interacting domains (RID) of MARTX toxins acylate the C-terminus of Rho GTPases as well [192]. Interestingly, the RIDs adopt an inverted papain-like fold [192], as predicted for IcsB [193]. The perturbation of Rho function through acylation might be extendable to other putative IcsB substrates [164], such as the septins [186], as well as Cdc42 and other actin-associated proteins implicated in the formation of actin cocoons [191]. Nevertheless, it is not clear if IcsB acylates all of these proteins and to what this might avail. Intriguingly, the cell-to-cell spread phenotype of Δ*icsB* appeared to be solely due to another putative IcsB substrate named CHMP5, which is a poorly studied regulator of the endosome sorting complex required for transport III (ESCRT-III) (Figure 5). The mechanistic contribution of the ESCRT machinery in preventing vacuole escape in *Shigella* is unknown.

Despite this progress in our understanding of the function of IcsB, it is important to consider the role of other virulence factors in vacuole escape. This is underscored by the observation that nonpolar *icsB* mutants have a small spreading defect that is hardly detectable in the plaque formation assay [140,164,194]. It is also noteworthy that this phenotype may vary depending on the *Shigella* strain or the host cell model studied [185]. Moreover, Δ*virA* displayed a more robust recruitment of LC3 than Δ*icsB*, and Δ*icsB* Δ*virA* displayed a much stronger plaque formation defect than both single-locus mutants [140]. This double mutant was used to identify a V-ATPase-dependent xenophagic response [195] whose role during WT *Shigella* infection is currently unknown. Similar to IcsB, VirA prevents the recruitment of LC3 to secreting bacteria residing in the dissemination vacuole. VirA is a Rab-specific GTPase-activating protein (GAP) that allows *Shigella* to dampen LC3 recruitment. Specifically, inactivation of Rab1 by VirA inhibited the endoplasmic reticulum (ER) to Golgi trafficking [196]. Although Rab1 involment in the ER exit site was implicated in the early stage of autophagosomes formation through the mobilization of ATG9A vesicles [197,198], it is not clear how Rab1 is tied to the recruitment of LC3 to *Shigella*-containing vacuoles. It is plausible that Rab1, akin to CHMP5, is involved in the repair of ruptured vacuoles (Figure 5); this might proceed through Rab1-mediated extension of the autophagosome membrane as suggested in macroautophagy [198].

Guanylate-binding proteins are a family of dynamin-related GTPases expressed in epithelial cells stimulated with interferon-γ (IFNγ). Through a C-terminal triple arginine motif, GBPs bind to the surface of *S. flexneri*, which, however, uses its effector IpaH9.8 to break free from GBPs coating [165,199,200,201]. Indeed, IpaH9.8 is an E3 ubiquitin ligase that can synthesize polyubiquitin K48 chains on several GBPs, thus degrading them through the proteasome [165,199]. GBP1 seems to be the most important target, as it is efficiently polyubiquitinated [165,199], and in its absence, other family members are not recruited on the bacterial surface [200]. In *Salmonella*, GBP1 is recruited to the surface of bacteria found in ruptured vacuoles [202]. Similar observations, although complicated by cytosolic movement, were also reported in *Shigella* [199]. These data suggest that IpaH9.8 acts downstream of vacuoles rupture just as IcsB and VirA do (Figure 5). How GBP restricts cell-to-cell spread and whether this leads to capture of *Shigella* into xenophagic vacuoles is unknown. The outer-membrane-associated *Shigella* protease IcsP was suggested to protect *Shigella* from complement C3-induced autophagy [203]. Taken together, these studies highlight the possibility that other virulence factors might be implicated in the escape of *Shigella* from xenophagy in specific immunological contexts.

## 6. Conclusions

*Shigella* is arguably one of the gold standard models for the T3SS. Since the early 2000s, several research groups have used it to describe the structure of the T3SA with impact well beyond this pathogen. The sophistication of our understanding of its structure and of the regulatory cascade that controls its expression in *Shigella* have little equivalent in bacteriology. Nonetheless, there are still many interesting phenomena to decipher. For example, the assembly and, particularly, the functioning of the T3SA remains opaque. We have no grasp of the motion of the T3SA during the secretion process. The role of the proton motive force and its eventual coupling with the ATPase have yet to be elucidated and pose questions. Our capacity to monitor the activity of the T3SA is somewhat limited by a detection lag due to the production and maturation of reporter proteins in transcription-based assays or by the low signal amplification of most direct secretion assays. The development of new approaches to address this issue is instrumental to tackling unresolved questions about bacterial pathogenesis. Finally, the ballet of host factors and effectors around the dissemination vacuole is another hot area. The cooperation of effectors, the meaning of their functional redundancies, and their diverging role in various cell types or immunological contexts are passionating topics for the years to come.
